# Implementing Doctor-Led DASA-IV to Inform MDT Risk Assessment on an Acute Adult Ward

**DOI:** 10.1192/bjo.2026.11622

**Published:** 2026-06-30

**Authors:** Mohammad Mansoor, Marion Joseph, Harith Ali, Areej Rasheed

**Affiliations:** East London NHS Foundation Trust, London, United Kingdom

## Abstract

**Aims:**

The Dynamic Appraisal of Situational Aggression (DASA-IV) is a brief, structured tool designed to assess short-term risk of violence in psychiatric inpatients. Whilst it has been used by nursing staff in some settings, evidence regarding the impact of systematic implementation and the role of medical staff in initiating its use remains limited.

This audit evaluated the impact of introducing routine, doctor-completed DASA-IV assessments on a general adult inpatient ward, with the intention of presenting findings to nursing staff and informing potential ward-wide implementation to support incident reduction and patient safety.

The primary aim was to assess whether weekday doctor-led completion of the DASA-IV was associated with changes in recorded agitation, aggressive incidents, and observation levels on the ward.

The secondary aim was to assess the feasibility and potential value of routine DASA-IV use as a precursor to broader nursing-led implementation.

It was hypothesised that consistent doctor-completed DASA-IV assessments would be associated with a reduction in aggression-related incidents and indicators of elevated clinical risk.

**Methods:**

A two-phase case-note review was conducted over four consecutive weeks on a general adult inpatient ward.

During the first two weeks (baseline phase), weekday nursing documentation was retrospectively reviewed to capture episodes of agitation, recorded aggressive incidents, andobservation levels, with no change to usual care.

During the subsequent two weeks (intervention phase), the ward doctor completed the DASA-IV daily on weekdays alongside routine clinical work, while the same outcome measures continued to be collected.

Data from the baseline and intervention phases were compared to explore changes temporally associated with the introduction of doctor-led DASA-IV assessments. Data collection was restricted to weekdays in both phases.

**Results:**

Across 84 patient-days (baseline n=44; intervention n=40), incident-day rates were similar (11.4% vs 10.0%), as were agitation-days (13.6% vs 12.5%). One-to-one observations occurred on one baseline day and none during intervention.

Forty weekday DASA-IV assessments were completed; scores were low overall (mean 0.7, median 0, range 0–5). Using standard thresholds, 80% were low risk, 15% moderate and 5% high. All incident- and agitation-days occurred with DASA-IV ≥2 (100% sensitivity). Specificity was 88.9% for incidents and 91.4% for agitation, with negative predictive values of 100%.

**Conclusion:**

Doctor-led weekday DASA-IV use was feasible and provided actionable risk stratification, with no events on low-risk days. Over this short audit, it was not associated with reduced incidents or agitation. Larger, longer evaluations with planned nursing-led implementation are needed.

